# Synthesis of Multicolor Core/Shell NaLuF_4_:Yb^3+^/Ln^3+^@CaF_2_ Upconversion Nanocrystals

**DOI:** 10.3390/nano7020034

**Published:** 2017-02-07

**Authors:** Hui Li, Shuwei Hao, Chunhui Yang, Guanying Chen

**Affiliations:** 1 MIIT Key Laboratory of Critical Materials Technology for New Energy Conversion and Storage, School of Chemistry and Chemical Engineering & Key Laboratory of Micro-systems and Micro-Structures, Ministry of Education, Harbin Institute of Technology, Harbin 150001, China; huili@hit.edu.cn (H.L.); yangchh@hit.edu.cn (C.Y.); 2 Institute for Lasers, Photonics and Biophotonics, State University of New York at Buffalo, Buffalo, NY 14260, USA

**Keywords:** core/shell nanocrystals, liquid-solid-solution method, thermal decomposition, multicolor emissions

## Abstract

The ability to synthesize high-quality hierarchical core/shell nanocrystals from an efficient host lattice is important to realize efficacious photon upconversion for applications ranging from bioimaging to solar cells. Here, we describe a strategy to fabricate multicolor core @ shell α-NaLuF_4_:Yb^3+^/Ln^3+^@CaF_2_ (Ln = Er, Ho, Tm) upconversion nanocrystals (UCNCs) based on the newly established host lattice of sodium lutetium fluoride (NaLuF_4_). We exploited the liquid-solid-solution method to synthesize the NaLuF_4_ core of pure cubic phase and the thermal decomposition approach to expitaxially grow the calcium fluoride (CaF_2_) shell onto the core UCNCs, yielding cubic core/shell nanocrystals with a size of 15.6 ± 1.2 nm (the core ~9 ± 0.9 nm, the shell ~3.3 ± 0.3 nm). We showed that those core/shell UCNCs could emit activator-defined multicolor emissions up to about 772 times more efficient than the core nanocrystals due to effective suppression of surface-related quenching effects. Our results provide a new paradigm on heterogeneous core/shell structure for enhanced multicolor upconversion photoluminescence from colloidal nanocrystals.

## 1. Introduction

Upconversion nanocrystals (UCNCs) are able to convert two or more long wavelength photons into short wavelength emissions through the use of real energy levels of trivalent lanthanide ions embedded in an inorganic host lattice [1]. Owing to the high physicochemical stability and intrinsic low phonon energy, fluoride-based UCNCs are able to minimize energy losses at the intermediate states of the incorporated lanthanide ions, thus generally exhibiting efficient upconversion (UC) luminescence efficiency [2]. Moreover, fluoride UCNCs also have superior features, such as low toxicity, non-blinking, non-photobleaching, absence of autofluorescence, and tissue-penetrable near-infrared (NIR) light excitation [3,4]. These superb attributes promise their applications in biological imaging [5,6,7,8], bio-detection [9,10], and three-dimensional display [11,12,13]. This motivation fuels a range of works to synthesize UCNCs with controlled size and morphology, as well as to prepare epitaxial core/shell UCNCs with enhanced efficiency and multifunction for theranostic applications, such as, NaYF_4_:Yb/Er@NaYF_4_ [14], (NaLuF_4_:Gd^3+^/Yb^3+^/Er^3+^)@NaLuF_4_:Yb^3+^ [15], and NaYbF_4_:Er@NaGdF_4_ core/shell nanostructures [16].

Sodium lutetium fluoride (NaLuF_4_) has recently emerged as a new type of efficient host lattice for photon upconversion, similar to the well-established host material of sodium yttrium fluoride (NaYF_4_). NaLuF_4_-based UCNCs have been demonstrated to exhibit bright upconversion luminescence (UCL) [17,18,19,20,21,22,23,24] and show efficient five- and four-photon ultraviolet emissions under continuous wave excitation at 980 nm [25,26,27]. Despite recent success in synthesizing NaLuF_4_ nanoplates or nanorods with size over ~30 nm using thermal decomposition method [28,29,30,31] or hydrothermal method [32,33,34,35,36,37,38], the ability to prepare uniform single crystal phase sub-10 nm NaLuF_4_ UCNCs remains elusive. Moreover, doping of a high concentration of inert Gd^3+^ ions (≥20%) was typically required to prepare small-sized monodisperse NaLuF_4_ UCNCs with single crystal phase previously [17,39], thus delivering traits actually from an entity of Lu-Gd alloyed host. On the other hand, small-sized UCNCs are important for single molecule imaging [40] and in vivo bioimaging with reduced toxicity, considering the renal clearance [41]. However, they always come at the sacrifice of UCL efficiency due to the increased size-induced surface-related surface quenching effect [42]. A core/shell geometric structure is therefore needed to eliminate or suppress this detrimental effect by spatial isolation of the core nanoparticle from the surrounding quenching sites. A straightforward approach is to grow a homogenous core/shell structure where the host of the shell is identical to the core [37,43]. However, the possible leaking of rare earth ions from the host lattice could possibly lead to diseases such as nephrogenic systemic fibrosis [44,45]. Compared with lanthanide fluorides, calcium fluoride (CaF_2_) has unique advantages owing to its superior biocompatibility and high optical transparency [46,47,48,49,50,51]. It has recently been demonstrated that CaF_2_ also has low lattice mismatch with NaReF_4_ nanocrystals, and can efficiently prevent rare-earth ions from leaking [46,50]. This implies that the growth of a CaF_2_ shell not only renders UCL enhancement, but also imparts biocompatibility with reduced leaking effect.

In this work, we describe our effort toward the controlled synthesis of single crystal phase sub-10 nm α-NaLuF_4_:Yb^3+^/Ln^3+^ (Ln = Er, Ho, or Tm) nanoparticles using a liquid-solid-solution method without the involvement of doping with a high concentration of Gd^3+^, and then utilize them as the core to epitaxially grow a high quality α-NaLuF_4_:Yb^3+^/Ln^3+^@CaF_2_ (Ln = Er, Ho, or Tm) core/shell UCNC via a thermal decomposition protocol. We found that the growth of a ~3 nm thin CaF_2_ shell layer was able to enhance the multicolour UCL of the core nanocrystals by up to ~772-fold.

## 2. Results and Discussion

### 2.1. Synthesis of α-NaLuF_4_:Yb^3+^/Ln^3+^ (Ln = Er, Tm, Ho) or α-NaLuF_4_:Yb^3+^/Ln^3+^@CaF_2_ Core/Shell UCNCs

**Figure 1:**
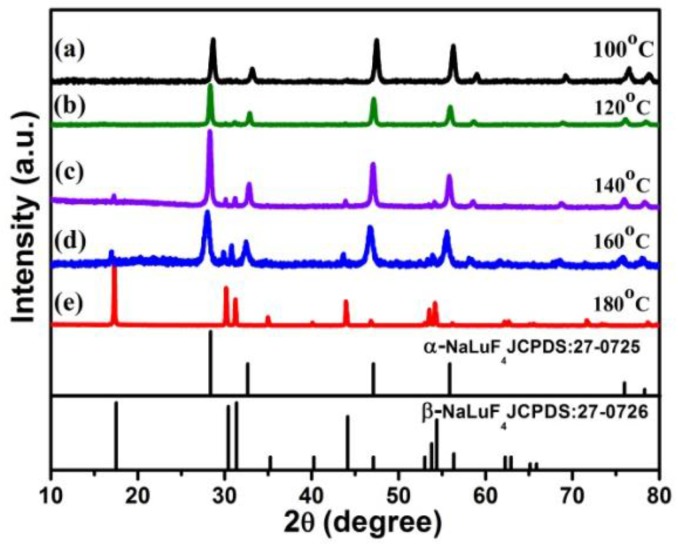
The X-ray diffraction patterns of NaLuF_4_:Yb^3+^/Ln^3+^ nanocrystals synthesized at different hydrothermal temperature: (a) 100 °C; (b) 120 °C; (c) 140 °C; (d) 160 °C; (e) 180 °C. All samples were prepared at F^−^/Ln^3+^ = 4:1.

The crystal structure of NaLuF_4_ has two forms of the cubic (α-) and the hexagonal (β-) phase. To prepare high-quality α-NaLuF_4_@CaF_2_ core/shell NCs, we firstly controlled the synthesis of cubic (α) NaLuF_4_ core nanoparticles by varying the reaction temperature and the molar ratio of F^−^/Ln^3+^ (Ln = Lu + Yb + Er/Ho/Tm) precursor. Figure 1 shows X-ray diffraction (XRD) patterns of NaLuF_4_:Yb^3+^/Er^3+^ (Tm^3+^, Ho^3+^) NCs hydrothermally prepared at various temperatures with the molar ratio of F^−^/Ln^3+^ (Ln = Lu + Yb + Er/Ho/Tm) fixed at 4:1. Figure 1 reveals that the phase transition process (β → α or α → β) occurs at a reaction temperature of *T* = 100 or 120 °C. The sample obtained at low temperature (*T* = 100 or 120 °C) shows nearly pure α-phase (JCPDS No. 27-0725). The diffraction peaks of β-NaLuF_4_ appear at temperatures between 140 and 160 °C (See Figure 1c,d), while at 180 °C, only pure β-phase NaLuF_4_ exists. It could be concluded that low temperature favors the formation of pure α-NaLuF_4_ core NCs.

**Figure 2:**
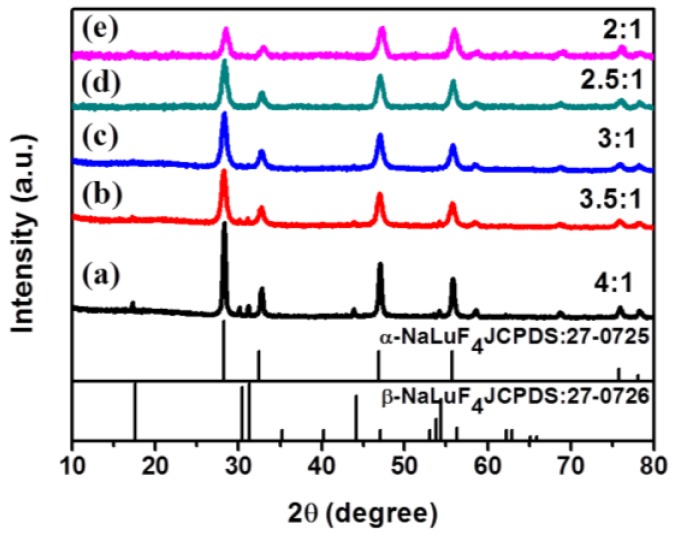
The X-ray diffraction patterns of NaLuF_4_:Yb^3+^/Er^3+^ nanocrystals synthesized with molar ratio of F^−^/Re^3+^ = (a) 4:1; (b) 3.5:1; (c) 3:1; (d) 2.5:1; (e) 2:1. (*T* = 140 °C).

**Figure 3:**
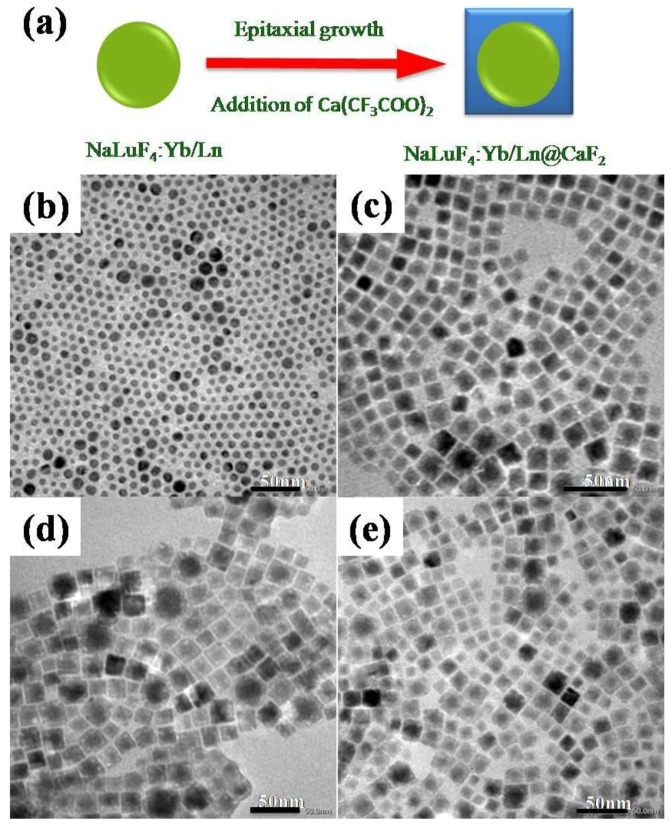
(**a**) Schematic illustration of the epitaxial growth of CaF_2_ shell on α-NaLuF_4_:Yb^3+^/Ln^3+^ core nanoparticles (NPs); (**b**) TEM image of α-NaLuF_4_:Yb^3+^/Ln^3+^ core nanocrystals prepared at F^−^/Re^3+^ = 3:1 and *T* = 140 °C. TEM images of (**c**) NaLuF_4_:Yb^3+^/Er^3+^@CaF_2_ core/shell upconversion nanocrystals (UCNCs); (**d**) α-NaLuF_4_:Yb^3+^/Ho^3+^@CaF_2_ core/shell UCNCs; (**e**) α-NaLuF_4_:Yb^3+^/Ln^3+^@CaF_2_ core/shell UCNCs. Particles in (c–e) were synthesized by the thermal decomposition method.

Next, we investigated the role of the molar ratio of F^−^/Ln^3+^ (Ln = Lu + Yb + Er/Ho/Tm) precursor on the crystal phase of our product when setting the synthesis temperature at 140 °C. As can be seen in Figure 2, when the molar ratio of F^−^/Ln^3+^ is fixed at 4:1, the sample shows a mixture of α-phase (JCPDS No. 27-0725) and β-phase (JCPDS No. 27-0726). As the F^−^/Ln^3+^ ratio is reduced to 3.5:1, the intensities of peaks of β-NaLuF_4_ decreases (Figure 2b). A pure α-phase NaLuF_4_ NCs can be produced at the ratio of F^−^/Re^3+^ below 3:1, and the diffraction peaks of α-NaLuF_4_ sample at F^−^/Re^3+^ = 3:1 show stronger intensity than at F^−^/Re^3+^ = 2.5:1 and 2:1. The average crystallite size of the nanocrystals was calculated according to Scherrer’s equation [51],

(1) D=Kλ/βcosθ

where *K* = 0.89, D represents the crystallite size (in nanometers), λ is the wavelength of the Cu Kα radiation, β is the corrected half-width of the diffraction peak, and θ is Bragg’s angle of the diffraction peak. According to Equation (1) and the half width of the main diffraction peak at 28° in Figure 2, the average size was calculated to be about 10 nm for F^−^/Re^3+^ = 3:1, in good agreement with the TEM result (Figure 3b). As a consequence, we selected α-NaLuF_4_ nanoparticles prepared at F^−^/Re^3+^ = 3:1 and *T* = 140 °C to epitaxially grow the α-NaLuF_4_:Yb/Ln@CaF_2_ core/shell structure.

The principle for the epitaxial growth of the core/shell structure is illustrated in Figure 3a, which involves an injection of the (CF_3_COO)_2_Ca solution into the growing solution containing pre-synthesized α-NaLuF_4_:Yb/Ln core nanocrystals prepared at F^−^/Re^3+^ = 3:1 and *T* = 140 °C. The morphologies and sizes of the α-NaLuF_4_:Yb^3+^/Ln^3+^ core and the resulting α-NaLuF_4_:Yb^3+^/Ln^3+^@CaF_2_ core/shell nanoparticles were examined by transmission electron microscopy (TEM), and the results are shown in Figure 3b. As one can see, the α-NaLuF_4_:Yb^3+^/Ln^3+^ core has sphere-like morphology with a size of 9 ± 0.9 nm, in good agreement with the XRD result in Figure 2c.

After growing the CaF_2_ shell, the core/shell NCs showed a cubic morphology with a size of 15.6 ± 1.2 nm (Figure 3c–e). The size difference indicated that a CaF_2_ shell with a thickness of about 3.3 ± 0.3 nm was grown on the surface of α-NaLuF_4_:Yb^3+^/Ln^3+^ core. Moreover, the formation of core/shell structure can be seen in the TEM images, shown in Figure 3c–e.

**Figure 4:**
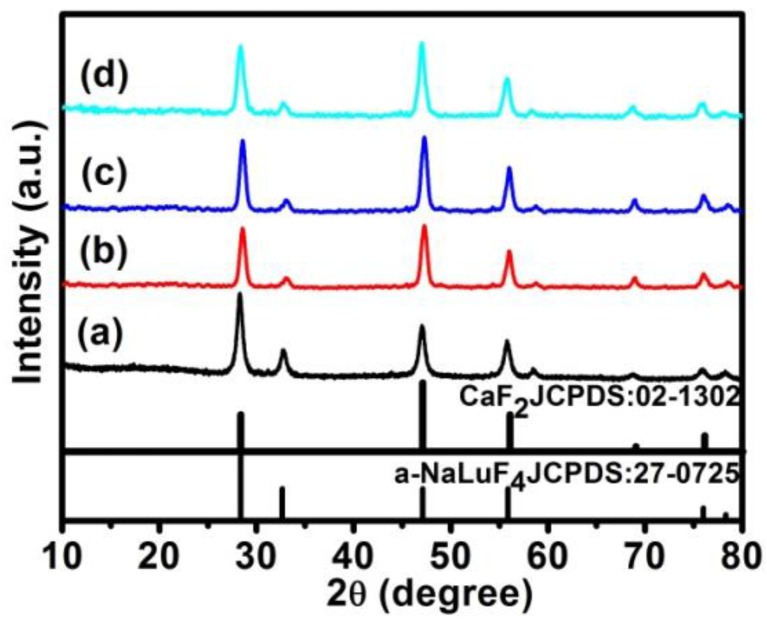
The X-ray diffraction patterns of the (a) α-NaLuF_4_:Yb^3+^/Ln^3+^ NCs; (b) α-NaLuF_4_:Yb^3+^/Er^3+^@CaF_2_ core/shell UCNCs; (c) α-NaLuF_4_:Yb^3+^/Ho^3+^@CaF_2_ core/shell UCNCs; and (d) the α-NaLuF_4_:Yb^3+^/Tm^3+^@CaF_2_ core/shell UCNCs, in reference to the standard diffraction patterns of the α-phase NaLuF_4_ (JCPDS 27-0725) and cubic phase CaF_2_ (JCPDS 02-1302).

The XRD peaks of the α-NaLuF_4_:Yb^3+^/Ln^3+^ core, the α-NaLuF_4_:Yb^3+^/Er^3+^@CaF_2_ core/shell, the NaLuF_4_:Yb^3+^/Ho^3+^@CaF_2_ core/shell, and the NaLuF_4_:Yb^3+^/Tm^3+^@CaF_2_ core/shell UCNCs are presented in Figure 4. As one can see, the core and core/shell NCs have identical peak positions, agreeing well with the standard JCPDF27-0725 sample of cubic NaLuF_4_ and JCPDF 02-1302 sample of cubic CaF_2_. The well-defined peaks are indicative of the high crystallinity of both the core and the core/shell NCs. The narrower XRD peaks of the core/shell NCs compared to that of core NPs indicate the larger size of core/shell NCs, in accordance with the TEM results in Figure 3.

### 2.2. Characterization of α-NaLuF_4_:Yb^3+^/Ln^3+^@CaF_2_ (Ln = Er, Tm, or Ho) Core/Shell UCNCs

**Figure 5:**
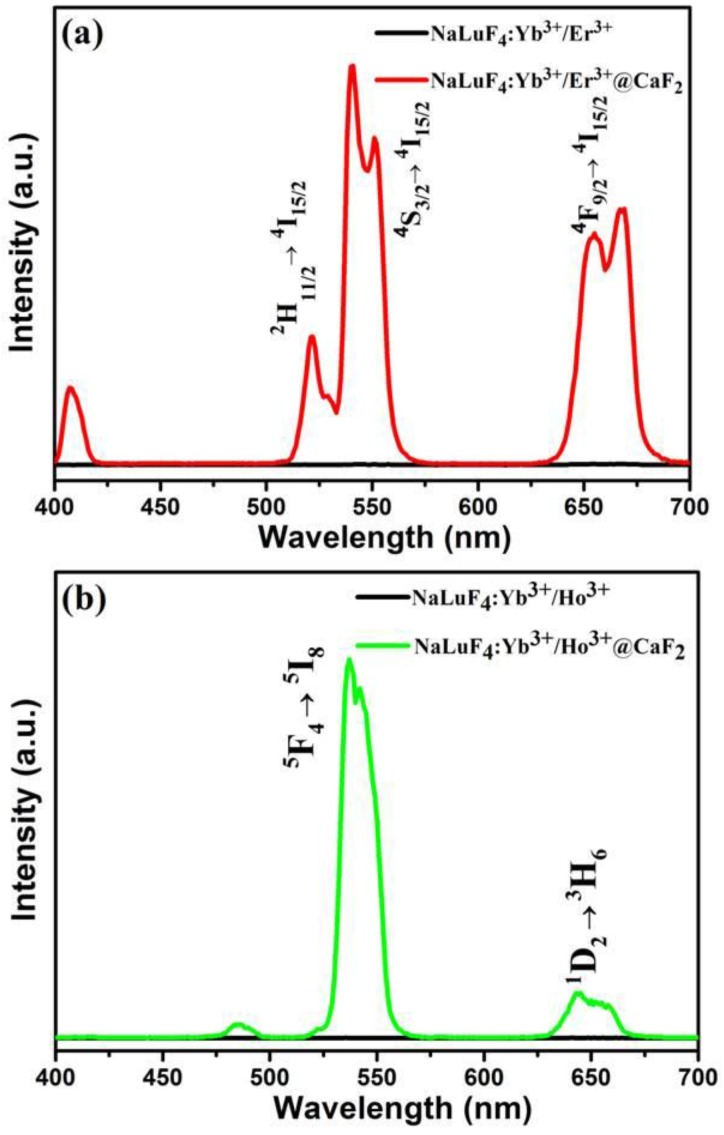

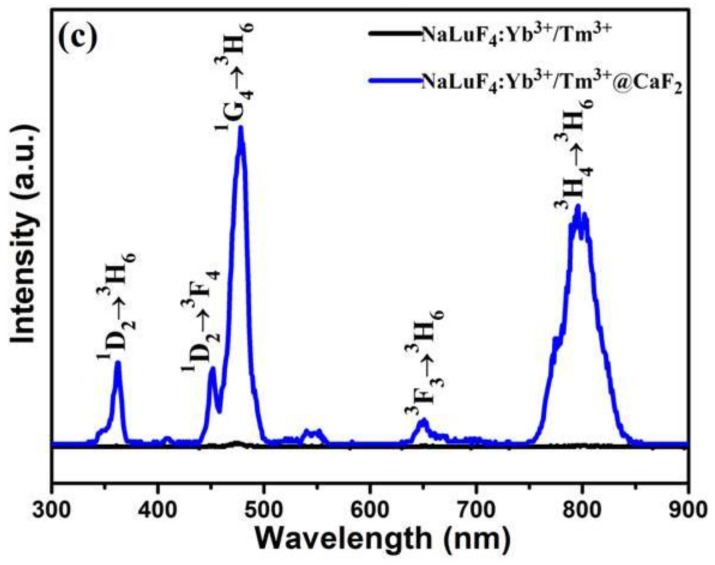
The upconversion luminescence spectra under excitation at 976 nm using a fiber-coupled laser diode: (**a**) the α-NaLuF_4_:Yb^3+^/Er^3+^ core and α-NaLuF_4_:Yb^3+^/Er^3+^@CaF_2_ core/shell NPs; (**b**) the α-NaLuF_4_:Yb^3+^/Ho^3+^ core and α-NaLuF_4_:Yb^3+^/Ho^3+^@CaF_2_ core/shell NPs; (**c**) the α-NaLuF_4_:Yb^3+^/ Tm^3+^ core and α-NaLuF_4_:Yb^3+^/Tm^3+^@CaF_2_ core/shell NPs. The concentration of Ln^3+^ in all samples was kept identical at about 0.5 mmol nanoparticles (i.e., nanoparticles formed by 0.5 mmol lanthanide precursors) per 10 mL cyclohexane.

We compared the UCPL from the corresponding core NaLuF_4_:20%Yb^3+^/2%Ln^3+^ and the core/shell NaLuF_4_:20%Yb^3+^/2%Ln^3+^@CaF_2_ UCNCs (Ln = Er, Ho, and Tm) (Figure 5). As shown in Figure 5a, two UC bands with maxima at 525/540 nm and 660 nm were observed from both the NaLuF_4_:20%Yb^3+^/2%Er^3+^ core and the NaLuF_4_:20%Yb^3+^/2%Er^3+^@CaF_2_ core/shell UCNCs, corresponding to the ^2^H_11/2_/^4^S_3/2_ → ^4^I_15/2_ and the ^4^F_9/2_ → ^4^I_15/2_ transitions of Er^3+^ ions, respectively. Moreover, the intensity of UCPL from core/shell UCNCs is dramatically higher than that of core nanoparticles. The intensity for UCPL band at 540 nm from the NaLuF_4_:20%Yb^3+^/2%Er^3+^@CaF_2_ core/shell NCs were found to be about 656 times higher than that from the α-NaLuF_4_:Yb^3+^/Er^3+^ core nanocrystals. Moreover, according to Figure 5b,c, when compared with the α-NaLuF_4_:Yb^3+^/Ho^3+^ and α-NaLuF_4_:Yb^3+^/Tm^3+^ core nanocrystals, the intensity of UCPL from the corresponding core/shell UCNCs showed a 772- and 75-fold enhancement, respectively. Taken together, we conclude that the core/shell structure resulted in a significant UCPL enhancement of the core nanocrystals. Since the CaF_2_ is an inactive layer, such an impressive enhancement undoubtedly arises from the suppression of surface quenching effects on the surface of core nanoparticles. In fact, the ultrasmall size (9 nm) of the core UCNCs is able to expose most of the doped sensitizer (Yb^3+^) and the doped activator (Er^3+^/Ho^3+^/Tm^3+^) to surface quenching effects (surface defects, surface strains, ligand and solvent molecules with groups possessing high vibration energy) [42] due to the extremely high “surface-to-volume” ratio. The epitaxial growth of the thin CaF_2_ shell not only eliminates the quenching defects on the surface of the core, but also shields all the sensitizer and activator ions in the core from the environmental quenching factors.

**Figure 6:**
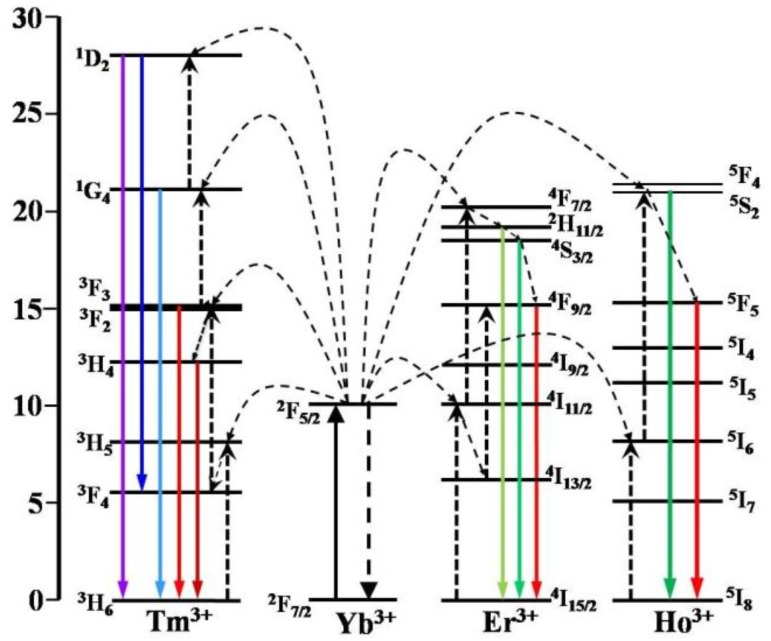
The energy level diagrams of Yb^3+^, Er^3+^, Tm^3+^, and Ho^3+^ ions, showing the proposed upconversion mechanisms in the α-NaLuF_4_:Yb^3+^/Ln^3+^@CaF_2_ (Ln = Er, Ho, or Tm) core/shell UCNCs.

To illustrate the UC mechanisms of the NaLuF_4_:20%Yb^3+^/2%Ln^3+^@CaF_2_ (Ln^3+^ = Er^3+^, Ho^3+^, Tm^3+^) NCs, possible UC processes are schematically given in the energy level diagrams of Yb^3+^, Er^3+^, Ho^3+^, and Tm^3+^ ions in Figure 6. The observed green UC bands (^2^H_11/2_ → ^4^I_15/2_, 525 nm; ^4^S_3/2_ → ^4^I_15/2_, 540 nm) and red UC band (^4^F_9/2_ → ^4^I_15/2_, 660 nm) from Er^3+^ ions in NaLuF_4_:20%Yb^3+^/2%Er^3+^@CaF_2_ UCNCs may take place via the following process: Yb^3+^ ion absorbs one laser photon and gets excited from the ground ^2^F_7/2_ state to the exclusive excited ^2^F_5/2_ state. The Yb^3+^ ions in the excited state transfer their absorbed energy to neighboring Er^3+^ ions and excite them from the ground ^4^I_15/2_ state to the ^4^I_11/2_ state, then to the ^4^F_7/2_ state. Multiphonon assisted relaxations from the ^4^F_7/2_ state can decay nonradiatively to the lower ^2^H_11/2_ and ^4^S_3/2_ levels, emitting the 525 and 540 nm UCL, respectively. The red emission 660 nm originates from the ^4^F_9/2_ → ^4^I_15/2_ transition, and the ^4^F_9/2_ state can be populated either from nonradiative relaxations from the ^4^S_3/2_ level or the energy transfer from Yb^3+^ ions to the Er^3+^ ion at the ^4^I_13/2_ state. In addition, the processes for green (^5^F_4_ → ^5^I_8_, 537 nm) and red (^5^F_5_ → ^5^I_8_, 645 nm) UCL of Ho^3+^ ions involved two different centers—the sensitizer (Yb^3+^), the activator (Ho^3+^)—along with two successive transfers from Yb^3+^ ions to Ho^3+^ ions. In the first transfer, the Yb^3+^ ion absorbs the excitation photons through the ground state absorption and transfers its absorbed energy to the neighboring Ho^3+^ ion to populate to its intermediate (^5^I_6_ level). The energy difference between the two levels was abridged by the vibration energy of the host lattice. The second transfer is to promote the population from the intermediate ^5^I_6_ level to the emitting energy levels (^5^S_2_) by energy transfer (ET) from another excited Yb^3+^ ion [52]. Once the ^5^S_2_ level is populated, the excited electron can release its energy by emitting green emissions. The red emission at 645 nm can be produced by radiative decay to the ground ^5^F_5_ state. The blue UCL of Tm^3+^ occurs via a three-step ET from Yb^3+^ to Tm^3+^. First, the Tm^3+^ ion in the ground state ^3^H_6_ is excited to the state ^3^H_5_ via an ET from a neighboring excited Yb^3+^ ion. Subsequent nonradiative relaxation of ^3^H_5_/^3^F_4_ populates the ^3^F_4_ level. In the second-step excitation, the Tm^3+^ ion in the ^3^F_4_ state is excited to the ^3^F_2,3_ state via another ET from a neighboring excited Yb^3+^ ion. The populated ^3^F_2_ level may nonradiatively relax to the ^3^F_3_ level. When the Tm^3+^ ion at the ^3^F_3_ level decays to the ground state, a weak red emission (^3^F_3_ → ^3^H_6_) is produced. Additionally, the near-infrared UCL at 802 nm arises from the ^3^H_4_ → ^3^H_6_ transition, where the ^3^H_4_ state is populated by the efficient nonradiative relaxation from the ^3^F_2,3_ state. A third energy transfer from Yb^3+^ excites Tm^3+^ at the ^3^F_3_ level to the ^1^G_4_ level, from which the blue emission (^1^G_4_ → ^3^H_6_) occurs by radiative decay to the ground state. The fourth energy transfer from Yb^3+^ promotes the Tm^3+^ at the ^1^G_4_ level to the ^1^D_2_ level, from which a 360 nm ultraviolet UCL (^1^D_2_ → ^3^H_6_) is generated.

## 3. Materials and Methods

### 3.1. Materials

All Ln_2_O_3_ (99.9%, Ln = Lu, Yb, Er, Tm, Ho) were obtained from Jianfeng Rare-Earth Limited Company, Conghua, China. The basic chemical reagents, such as sodium hydroxide, oleic acid (OA), absolute ethyl alcohol, trifluoroacetic acid (TFA), calcium oxide, sodium fluoride and octadecene (ODE) were purchased from Sinopharm Chemical Reagent Co., Ltd., Beijing, China. All chemicals were of analytical grade and were used as received without further purification.

### 3.2. Hydrothermal Synthesis of α-NaLuF_4_:Yb^3+^/Ln^3+^ UCNCs

We synthesized α-NaLuF_4_:Yb^3+^/Ln^3+^ (Ln = Er, Tm, or Ho) nanocrystals by a hydro-thermal method adapted from the literature [53]. Typically, 0.6 g of NaOH, 3 mL of water, 14 mL of oleic acid (OA) (90 wt. %), and 10 mL (120 mmol) of ethanol were well-mixed at room temperature to yield a white viscous solution. Then, 1 mmol Ln(NO_3_)_3_ (Ln = Lu, Yb, Er, Tm, Ho, and Lu^3+^:Yb^3+^:Er^3+^/Tm^3+^/Ho^3+^ = 78%:20%:2%/2%/2%) was added into the above solution and kept vigorous stirring. After aging for 30 min, 4 mL (4 mmol) of NaF (F^−^/Ln^3+^ = 4:1) solution was added under vigorous stirring for 30 min. Subsequently, the mixture was transferred to a 50-mL Teflon-lined autoclave and heated at 140–180 °C for 24 h. After washing with ethanol, the final products were dispersed in cyclohexane.

### 3.3. Thermal Decomposition Synthesis of α-NaLuF_4_:Yb^3+^/Ln^3+^@CaF_2_ Core/Shell UCNCs

The core/shell nanoparticles were synthesized using the thermal decomposition method. Typically, 0.5 mmol CaO was first added to a 50 mL flask containing 5 mL deionized water and 5 mL trifluoroacetic acid (TFA). The solution was heated at 90 °C until the solution became transparent, and was then dried at this temperature with nitrogen purge to yield the shell precursor (CF_3_COO)_2_Ca. After obtaining the (CF_3_COO)_2_Ca powders, 10 mL of OA, 10 mL of ODE, and the pre-prepared α-NaLuF_4_:Yb^3+^/Ln^3+^ (0.5 mmol) in cyclohexane were added. The solution was then vacuum-degassed at 120 °C for 30 min to remove water, oxygen, and cyclohexane. Subsequently, the solution was heated to 300 °C at a rate of 15 K·min^−1^ under nitrogen protection. After maintaining at 300 °C for 30 min, the reaction was stopped and cooled down to room temperature. After washing with ethanol, the products were dispersed in cyclohexane for further use.

### 3.4. Thermal Decomposition Synthesis of α-NaLuF_4_:Yb^3+^/Ln^3+^@CaF_2_ Core/Shell UCNCs

The size and morphology of the α-NaLuF_4_:Yb^3+^/Ln^3+^ and α-NaLuF_4_:Yb^3+^/Ln^3+^@CaF_2_ core/shell nanocrystals were characterized by transmission electron microscopy (TEM) using a JEOL JEM-2010 microscope (JEOL Ltd., Tokyo, Japan) at an acceleration voltage of 200 kV. The powder X-ray diffraction (XRD) patterns were recorded by a Siemens D500 diffractometer (Bruker Beijing Scientific Technology Co. Ltd, Beijing, China) using Cu Kα radiation (λ = 0.15418 nm). The 2θ angle of the XRD spectra was recorded at a scanning rate of 5°/min. The UCPL spectra were obtained using a Zolix monochoromator (Beijing Zolix Instruments CO., Ltd., Beijing, China) under excitation at 976 nm using a fiber-coupled laser diode (BWT Beijing Ltd., Beijing, China).

## 4. Conclusions

In summary, cubic phase α-NaLuF_4_:Yb/Ln cores can be precisely controlled through a simple variation of reaction temperature and the added amount of NaF in a hydrothermal method. Moreover, a seed-mediated growth protocol with selected parameters favorable for shell growth yields the α-NaLuF_4_:Yb/Ln@CaF_2_ (Ln = Er, Ho, Tm) core/shell structure NPs having a core size of ~9 nm and shell thickness of ~3.3 nm. Moreover, we found that the growth of the inert thin shell of CaF_2_ onto the α-NaLuF_4_:Yb/Ln (Ln = Er, Ho, Tm) core could enhance its multicolor UCL by up to 772-fold, being attributed to effective suppression of surface-related quenching effects via spatial isolation of the core from the surrounding environment. Small-sized α-NaLuF_4_:Yb/Ln@CaF_2_ (Ln = Er, Ho, Tm) UCNCs developed here have implication for uses in a range of biophotonic applications, such as bioimaging.
